# Effect of Zoledronic Acid on Skeletal Muscle After Bariatric Surgery: A Secondary Analysis From a Randomized Controlled Trial

**DOI:** 10.1002/oby.70062

**Published:** 2025-11-02

**Authors:** Søren Gam, Anne Pernille Hermann, Claus Bogh Juhl, Stinus Gadegaard Hansen, Bibi Gram

**Affiliations:** ^1^ Department of Endocrinology, Esbjerg Hospital University Hospital of Southern Denmark Esbjerg Denmark; ^2^ Steno Diabetes Center Odense Odense University Hospital Odense Denmark; ^3^ Department of Regional Health Research University of Southern Denmark Odense Denmark; ^4^ Department of Endocrinology Odense University Hospital Odense Denmark; ^5^ Department of Clinical Research University of Southern Denmark Odense Denmark; ^6^ Research Unit of Endocrinology: Bariatrics and Diabetes University Hospital of Southern Denmark Esbjerg Denmark

**Keywords:** antiresorptive, bariatric surgery, muscle atrophy, muscle function, obesity, zoledronic acid

## Abstract

**Objective:**

This study aimed to investigate whether a single infusion of 5 mg zoledronic acid, given before bariatric surgery to prevent bone loss, could also hinder the loss of muscle mass, strength, and physical function.

**Methods:**

In this double‐blinded study, patients referred for bariatric surgery were randomized 1:1 to either intervention (INT: single dose zoledronic acid 5 mg) or placebo (CON). Assessments were conducted at baseline and 12 months postoperatively. The outcomes were body composition (DXA), muscle strength for knee extensor (KE) and knee flexor (KF), and physical function.

**Results:**

Fifty‐nine patients (age: 48.9 ± 6.3 years, BMI: 42.3 ± 5.3 kg/m^2^) were allocated to INT (*n* = 31) or CON (*n* = 28). At 12 months, no between‐group differences were observed in body weight, fat mass, or lean body mass. Both groups experienced ~14% loss of lean body mass. No between‐group differences were observed for absolute or relative muscle strength. Absolute strength declined by 11%–18%, while relative strength improved by 10%–22%. No between‐group differences were found in physical function measures, all of which improved by 5%–18%.

**Conclusions:**

A single infusion of 5 mg zoledronic acid did not prevent the loss of muscle mass or strength or improve physical function.

**Trial Registration**: ClinicalTrials.gov identifier: NCT04742010; EudraCT number: 2019‐001650‐26

**Study Importance**

- What is already known?
  ○ Bariatric surgery induces substantial loss of muscle mass and strength, which may impair physical function and increase frailty
  ○ Zoledronic acid prevents bone loss and may also preserve skeletal muscle under muscle‐wasting conditions
  ○ The potential of bisphosphonates to protect muscle mass and function in humans after bariatric surgery remains to be explored
- What does this study add?
  ○ This study shows that zoledronic acid does not prevent the loss of muscle mass or muscle strength, nor does it improve physical function 12 months after surgery
- How might these results change the direction of research or the focus of clinical practice?
  ○ Future studies should explore alternative dosing strategies (e.g., repeated dosing, postoperative administration) or investigate other pharmacological or exercise‐based interventions to counteract muscle loss in this population.

## 1 Introduction

Bariatric surgery is the most effective method for treating obesity, improving obesity‐related comorbidities, and lowering mortality rates [1]. Roux‐en‐Y gastric bypass (RYGB) and sleeve gastrectomy (SG) are the most performed procedures, inducing an ~32% reduction in body weight within the first year after surgery [2].

Rapid weight loss following bariatric surgery leads to substantial loss of skeletal muscle tissue. The mechanism for this is multifactorial, including mechanical unloading, malnutrition (such as protein deficiency), and inadequate caloric intake [3]. Lean body mass (LBM) and fat‐free mass (FFM) are frequently used as surrogate measures for muscle mass [4]. Skeletal muscle is a major component of FFM, typically accounting for ~45% in MRI‐based measurement (the gold standard) among individuals with obesity, although this proportion varies, ranging from 40% to 50% [5]. Within the first year post surgery, studies have shown a loss of 14%–19% in FFM, LBM, and appendicular lean mass (ALM) [2, 3, 4], which represents the LBM in the arms and legs [3]. During weight loss, it is generally expected that approximately 25% of total weight loss consists of FFM [6]. However, following bariatric surgery, there is considerable variability in FFM loss among patients, with 7%–28% experiencing excessive FFM loss at 12 months post surgery, defined as a reduction of 25%–35% or more of FFM relative to total body weight lost [2, 3].

Excessive loss of skeletal muscle mass has several undesirable consequences. Skeletal muscle tissue is metabolically active, and excessive loss leads to a decrease in basal metabolic rate [7], contributing to weight regain following bariatric surgery [8]. Improvements in physical function and relative muscle strength (i.e., muscle strength relative to body weight) are commonly observed during the first 12 months after surgery [9, 10, 11], but these changes are generally considered to be primarily driven by the weight loss itself rather than actual improvements in muscle function. Conversely, muscle mechanical function is negatively affected, with 16%–23% reductions in absolute maximal isokinetic and isometric lower limb muscle strength within the first year post surgery [10, 11]. A decline in absolute muscle strength may be problematic, as it independently associates with a greater risk of frailty [12], cardiometabolic diseases, and an increase in mortality [13, 14, 15]. Therefore, it is important to implement effective intervention protocols aimed at maintaining muscle mass and strength in this population.

Bisphosphonates (e.g., risedronate, zoledronic acid) are used for the treatment of osteoporosis, and both frequent administration and a single administration of zoledronic acid have been shown to prevent bone loss and significantly reduce fracture risk [16, 17]. We have recently shown that zoledronic acid can be used as a prophylactic treatment for bone loss in patients who underwent bariatric surgery [18]. Bisphosphonates might have a protective effect on skeletal muscle tissue under muscle‐wasting conditions, as primarily observed in preclinical studies and in one clinical study involving bariatric surgery patients [19, 20, 21, 22, 23]. However, several human studies in older adults with age‐related muscle loss have not shown significant effects of bisphosphonates on muscle mass or strength [24, 25, 26, 27, 28]. The potential mechanism for bisphosphonates' muscle‐sparing effect might work through two distinct or complementary mechanisms. Firstly, bisphosphonates prevent abnormal osteoclastic bone resorption (as observed following bariatric surgery) and thereby prevent the release of the cytokines stored in the bone matrix, such as transforming growth factor β (TGFβ) and receptor activator of nuclear factor kappa‐B ligand (RANKL). TGFβ and RANKL play a direct role in causing muscle loss and weakness [21, 29, 30]. The second mechanism is that bisphosphonates may act directly on skeletal muscle tissue by preventing the activation of muscle atrophy pathways during muscle‐wasting conditions [20, 22].

In our study, we found that zoledronic acid prevented bone loss in patients undergoing bariatric surgery and may thereby have prevented the release of stored TGFβ and RANKL [18]. These findings, combined with a study showing that the bisphosphonate risedronate blunted muscle loss in patients undergoing bariatric surgery [23], indicate that bisphosphonates could be a potential treatment for mitigating the loss of muscle mass and strength in patients undergoing bariatric surgery. Therefore, we aimed to investigate whether zoledronic acid can be used as a prophylactic treatment not only with respect to bone loss but also to prevent the loss of LBM and muscle strength in patients undergoing bariatric surgery. We hypothesized that zoledronic acid will preserve muscle mass and function compared with a placebo group.

## 2 Methods

### 2.1 Study Design

The present study is a secondary analysis of a randomized, double‐blinded, single‐center study that investigated the effect of zoledronic acid for the prevention of bone loss following bariatric surgery, including assessment of safety and tolerability [18]. In this study, we specifically aimed to investigate the effect of zoledronic acid for the prevention of loss of lean body mass and muscle function. The study was carried out at the University Hospital of Southern Denmark, Esbjerg. A detailed description of the study protocol has been published [31]. The study was registered at ClinicalTrials.gov (NCT04742010) and approved by the Regional Committee on Health Research Ethics for Southern Denmark (project identifier S‐20190134) and the Danish Medicines Agency (Z0L6700). All planned outcomes were reported in accordance with the CONSORT guidelines. After enrollment and baseline assessment, we randomized the participants into two groups: the zoledronic acid group or the placebo group. According to the protocol, the study drug was to be administered between 59 and 7 days prior to surgery. However, due to COVID‐19‐related delays, 20% of participants exceeded this window, and the maximum interval was extended to 180 days, which was considered acceptable given the long‐lasting effects of zoledronic acid [17].

### 2.2 Participants, Randomization, and Blinding

All recruited participants were patients referred for bariatric surgery at the University Hospital of Southern Denmark, Esbjerg. All participants provided informed written consent. We randomized participants 1:1 to either zoledronic acid (INT) or placebo (CON) using block randomization (with block sizes of 2, 4, and 6) and stratified an equal number of participants undergoing RYGB or SG into each study arm. Details about inclusion and exclusion criteria, surgical procedures, blinding, sequence generation, allocation concealment, and implementation have been published elsewhere [31].

### 2.3 Intervention

We administered a single dose of the study drug (zoledronic acid 5 mg or placebo) intravenously in a solution containing 100 mL of isotonic saline over a period of ≥ 15 min. All participants received standard bariatric care in Denmark, including dietary counseling before surgery and at 1, 6, 12, and 24 months postoperatively.

### 2.4 Outcomes

#### 2.4.1 Body Weight and Composition

Height and body weight were measured using a stadiometer and calibrated scale. Fat mass, LBM, and ALM were determined by dual‐energy X‐ray absorptiometry (DXA) (Hologic Horizon A). LBM refers to the weight of all body components except for fat and bones and thus includes muscles, water, organs, and connective tissues [3].

#### 2.4.2 Muscle Strength for Lower and Upper Extremities

We measured both upper and lower extremity muscle strength. Detailed descriptions of the applied test protocol and reliability data have been published [32, 33, 34]. The reliability for the applied muscle strength assessment ranged from good to excellent (ICC: 0.85–0.97) [32, 33].

For the lower extremities, we assessed the isokinetic and isometric knee flexor/extensor (KF/KE) [32] and ankle dorsi/plantar flexor (DF/PF) strength [33] of the nondominant leg using an isokinetic dynamometer (System 4, Biodex Medical System Inc.). For the upper extremities, we measured isometric shoulder elevation strength using a Bofors MODEL dynamometer (Bofors Elektronic) mounted in a reproducible standardized setup [35], and for maximal handgrip strength (HGS) of the nondominant arm, we used a handheld dynamometer (Jamar Plus, Patterson Medical) [36]. The participants performed three to five maximal attempts separated by 30–60 s. For all muscle strength assessments, the participants were instructed to contract as “hard and fast” as possible, and verbal encouragement was provided during testing.

#### 2.4.3 Physical Function

We measured habitual and maximal walking speed, walking endurance capacity, and lower limb muscle power. We have previously published the test procedures and reliability data for the applied physical function tests, which demonstrated good‐to‐excellent reliability (ICC: 0.84–0.94) for all tests except for habitual walk speed (ICC: 0.54) [34].

For habitual and maximal walking speed, we used the 3‐min walk test (3MWT) and the 7.6‐min walk test (7.6MWT), respectively. Each participant performed two attempts, and the fastest attempt was recorded as walking speed (m/s).

For endurance capacity, we used a 2‐min walk test (2MWT). Participants were instructed to walk as far as possible on a 20‐m track for 2 min. Results were reported as mean walking speed (m/s).

For assessing lower limb power, we used the stair climb power test (SCPT) and the 5‐repetition sit‐to‐stand test (STS5). A detailed description of procedures and equations for the calculation of power (W and W/kg) can be found elsewhere [34].

#### 2.4.4 Physical Activity

We used the International Physical Activity Questionnaire—Short Form (IPAQ‐SF) to assess differences in self‐reported physical activity levels between the groups based on the following categories: walking, moderate‐intensity activity, high‐intensity activity, and total physical activity level.

### 2.5 Statistical Analysis

This study is a secondary analysis of a randomized controlled trial originally powered to detect changes in volumetric bone mineral density in the lumbar spine at 12 months. No formal power calculations were performed for the present secondary outcomes related to muscle strength and physical function. The study sample size of 42 participants was determined based on the expected change in the primary bone outcome. To allow for dropouts, we aimed to randomize 60 participants. The secondary outcomes have a flat outcome structure with multiple evenly valued outcome measures and not a specified hierarchy of outcomes as seen for confirmatory trials [37].

Prior to analysis, data normality was tested by the Shapiro–Wilk test and graphically displayed by *Q*–*Q* plots. In the case of a non‐normal distribution, data were transformed using logarithm or square root. After transformation, normality was tested again. If normality was still violated, a nonparametric test was used.

An independent *t*‐test was used to assess differences in baseline values between groups. The effect of zoledronic acid was assessed following the intention‐to‐treat principle. We employed a mixed‐effects model with repeated measures, incorporating fixed factors as group (INT or CON) and time, along with their interaction. For the adjusted analysis, the covariates age, sex, and surgery type (randomization factor) were included. Participants were treated as random factors to account for individual variability.

To analyze differences between surgery types, we employed a mixed‐effects model with repeated measures, incorporating fixed factors of type of surgery and time, along with their interaction, and included covariates of age and sex. All data are presented as mean ± standard deviation (SD) or 95% confidence interval (95% CI), and the significance level was set at *p* < 0.05.

## 3 Results

Fifty‐nine participants were included in the intention‐to‐treat analysis, and a detailed description of the participants' flow is published elsewhere [18].

At baseline, there were no differences between the two study groups regarding age, height, weight, BMI, LBM, fat mass, type of surgery, physical activity level, or the time of administration of the study medication prior to surgery (Table 1). However, there was a difference in sex distribution (more males assigned to INT), fat percentage, ALM, and several measures of muscle strength and physical function.

**TABLE 1:** Participant characteristics at baseline.

	*N*	INT (*n* = 31)	*N*	CON (*n* = 28)	*p*
		Mean ± SD		Mean ± SD	
Age (years)	31	48.5 ± 6.3	28	49.3 ± 6.5	0.603
Sex: F/M (*n*)	31	19/12	28	24/4	0.019
Height (cm)	31	172.3 ± 8.6	28	169.5 ± 8.4	0.236
Weight (kg)	31	124.6 ± 20.9	28	122.8 ± 14.4	0.703
BMI (kg/m^2^)	31	41.8 ± 5.6	28	42.8 ± 4.8	0.485
Surgery type: RYGB/SG (*n*)	31	20/11	28	20/8	0.590
Administration of the study medication prior to surgery (days)	31	26.3 ± 21.0	28	40.4 ± 46.2	0.401
Body composition					
Fat mass (kg)	31	53.5 ± 11.2	28	58.1 ± 9.1	0.089
Fat percentage (%)	31	43.0 ± 5.8	28	47.1 ± 4.5	0.004
LBM (kg)	31	67.0 ± 13.0	28	61.4 ± 8.6	0.058
ALM (kg)	31	31.0 ± 7.2	28	27.7 ± 4.9	0.047
Muscle strength					
Handgrip strength (kg)	30	40.7 ± 11.5	28	36.6 ± 11.2	0.173
Shoulder elevation strength (Nm)	30	131.0 ± 62.9	28	94.0 ± 36.1	0.009
KE 180°/s (Nm)	30	140.5 ± 44.1	27	123.8 ± 34.0	0.118
KE 180°/s (Nm/kg)	30	1.12 ± 0.27	27	1.00 ± 0.24	0.092
KE 75° (Nm)	30	248.4 ± 77.4	27	224.1 ± 64.1	0.206
KE 75° (Nm/kg)	30	1.98 ± 0.49	27	1.81 ± 0.46	0.200
KF 180°/s (Nm)	30	72.9 ± 25.8	27	61.1 ± 15.3	0.044
KF 180°/s (Nm/kg)	30	0.57 ± 0.14	27	0.50 ± 0.11	0.028
KF 30° (Nm)	30	110.6 ± 35.7	27	95.2 ± 22.3	0.059
KF 30° (Nm/kg)	30	0.88 ± 0.21	27	0.77 ± 0.17	0.048
PF 90°/s (Nm)	30	75.1 ± 21.4	27	67.3 ± 18.0	0.204
PF 90°/s (Nm/kg)	30	0.59 ± 0.14	27	0.59 ± 0.25	0.939
PF 0° (Nm)	30	118.1 ± 40.6	27	106.5 ± 24.6	0.204
PF 0° (Nm/kg)	30	0.94 ± 0.26	27	0.87 ± 0.23	0.304
DF 90°/s (Nm)	26	9.2 ± 6.9	23	5.0 ± 4.3	0.015
DF 90°/s (Nm/kg)	26	0.07 ± 0.05	23	0.05 ± 0.04	0.045
DF 20° (Nm)	29	24.1 ± 10.6	23	17.5 ± 7.9	0.016
DF 20° (Nm/kg)	29	0.19 ± 0.08	23	0.15 ± 0.07	0.026
Physical function test					
3MWT (m/s)	30	1.10 ± 0.18	28	1.03 ± 0.19	0.166
7.6MWT (m/s)	27	2.00 ± 0.34	28	1.75 ± 0.31	0.007
2MWT (m/s)	30	194.6 ± 26.4	28	177.8 ± 34.3	0.041
STS5 (W)	30	544.4 ± 184.6	28	458.3 ± 118.8	0.041
STS5 (W/kg)	30	4.3 ± 1.1	28	3.7 ± 0.8	0.026
SCPT (W)	30	629.5 ± 150.4	27	583.7 ± 161.0	0.271
SCPT (W/kg)	30	5.0 ± 0.9	27	4.7 ± 1.1	0.276

*Note: p* values denote significant differences between zoledronic acid and placebo groups.

Abbreviations: 2MWT, 2‐min walk test; 3MWT, 3‐min walk test; 7.6MWT, 7.6‐min walk test; ALM, appendicular lean mass; CON, placebo group (*n* = 28); DF, dorsiflexion; F, female; INT, zoledronic acid group (*n* = 31); KE, knee extension; KF, knee flexion; LBM, lean body mass; M, male; PF, plantar flexion; RYGB, Roux‐en‐Y gastric bypass; SCPT, stair climb power test; SG, sleeve gastrectomy; STS5, 5‐repetition sit‐to‐stand test.

### 3.1 Body Composition

**FIGURE 1:**
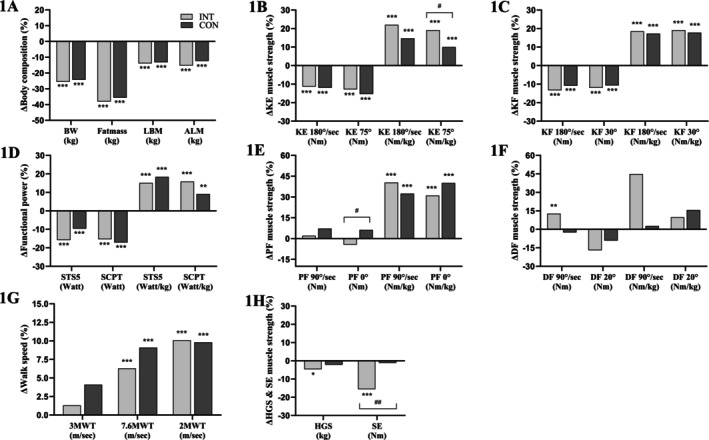
Within‐group relative changes in various body composition, muscle strength, and physical function metrics at 12 months post treatment. The light gray bars represent the zoledronic acid group, while the dark gray bars represent the control group. 2MWT, 2‐min walk test; 3MWT, 3‐min walk test; 7.6MWT, 7.6‐min walk test; ALM, appendicular lean mass; BW, body weight; CON, control group; DF, dorsiflexion; HGS, handgrip strength; INT, intervention group; KE, knee extension; KF, knee flexion; LBM, lean body mass; PF, plantar flexion; SCPT, stair climb power test; SE, shoulder elevation; STS5, 5‐repetition sit‐to‐stand test. Statistical significance between baseline and 12 months within group is indicated by *p < 0.05, **p < 0.01, ***p < 0.001. ^#^ indicates a significant interaction between the INT and CON, showing different changes over time between the two groups.

At the 12‐month follow‐up, there were no between‐group differences in the reduction of body weight, fat mass, LBM, or ALM (Table 2). Body weight, fat mass, LBM, and ALM decreased by 25%, 37%, 14%, and 14%, respectively (Figure 1, Panel A). The observed LBM loss accounted for 29% of the total weight loss.

**TABLE 2:** 12‐Month treatment effect estimates on body composition, muscle strength, and physical function.

	INT (*N* = 31)				CON (*N* = 28)				Between‐group differences	
	Baseline		12 month		Baseline		12 month		(INT – CON)	
	*N*	Mean (CI)	*N*	Mean (CI)	*N*	Mean (CI)	*N*	Mean (CI)	Mean (CI)	*p*
Body composition										
Weight (kg)	31	121.8 (116.1, 127.6)	29	**90.6 (84.9, 96.4)**	28	125.4 (119.5, 131.4)	24	**95.0 (88.7, 101.3)**	−0.7 (−6.1, 4.6)	0.789
BMI (kg/m^2^)	31	41.8 (39.9, 43.7)	29	**31.4 (29.5, 33.3)**	28	42.8 (40.9, 44.8)	24	**32.1 (30.0, 34.1)**	0.4 (−1.5, 2.2)	0.687
Fat mass (kg)	31	53.8 (50.1, 57.4)	29	**33.3 (29.5, 37.1)**	28	57.6 (53.7, 61.5)	24	**37.0 (32.9, 41.2)**	0.02 (−4.3, 4.4)	0.992
Fat percentage (%)	31	44.3 (42.7, 45.9)	29	**35.6 (33.9, 37.2)**	28	45.7 (44.0, 47.4)	24	**38.0 (36.1, 39.9)**	−1.0 (−3.7, 1.7)	0.458
LBM (kg)	31	64.0 (61.8, 66.2)	29	**55.1 (52.9, 57.4)**	28	64.5 (62.2, 66.8)	24	**56.0 (53.4, 58.4)**	−0.40 (−2.4, 1.5)	0.675
ALM (kg)	31	29.3 (28.2, 30.5)	29	**24.9 (23.7, 26.0)**	28	29.3 (28.1, 30.6)	24	**25.7 (24.4, 27.0)**	−0.86 (−1.9, 0.2)	0.179
Muscle strength										
HGS (kg)	30	37.3 (35.2, 39.5)	29	**35.6 (33.5, 37.8)**	28	39.8 (37.6, 42.0)	24	38.9 (36.6, 41.2)	−0.8 (−2.8, 1.2)	0.421
SE (Nm)	30	115.4 (105.2, 125.5)	27	**97.3 (87.1, 107.6)**	28	106.1 (95.9, 116.4)	24	104.9 (93.9, 115.9)	−16.8 (−29.1, −4.4)	0.008
KE 180°/s (Nm)	30	128.1 (121.2, 135.0)	29	**113.6 (106.7, 120.4)**	27	134.2 (127.1, 141.4)	23	**118.2 (106.7, 120.4)**	1.5 (−6.1, 9.2)	0.692
KE 180°/s (Nm/kg)	30	1.04 (0.97, 1.10)	29	**1.26 (1.20, 1.33)**	27	1.06 (0.99, 1.13)	23	**1.22 (1.15, 1.29)**	0.07 (−0.02, 0.17)	0.126
KE 75° (Nm)	30	227.2 (213.9, 240.5)	29	**198.0 (184.8, 211.3)**	27	242.3 (228.5, 256.1)	23	**205.1 (190.4, 219.7)**	8.1 (−6.4, 22.5)	0.273
KE 75° (Nm/kg)	30	1.84 (1.72, 1.97)	29	**2.19 (2.07, 2.32)**	27	1.93 (1.80, 2.06)	23	**2.12 (1.98, 2.26)**	0.16 (0.02, 0.29)	0.021
KF 180°/s (Nm)	30	67.6 (63.0, 72.3)	29	**58.6 (54.0, 63.3)**	27	66.0 (61.1, 70.9)	23	**58.8 (53.7, 64.0)**	−1.8 (−7.0, 3.4)	0.459
KF 180°/s (Nm/kg)	30	0.54 (0.51, 0.58)	29	**0.64 (0.61, 0.68)**	27	0.52 (0.49, 0.56)	23	**0.62 (0.57, 0.66)**	0.01 (−0.04, 0.14)	0.739
KF 30° (Nm)	30	103.8 (96.7, 110.9)	29	**91.3 (84.2, 98.4)**	27	98.2 (89.4, 106.9)	23	**91.0 (83.0, 98.9)**	−1.6 (−10.3, 7.1)	0.718
KF 30° (Nm/kg)	30	0.84 (0.78, 0.90)	29	**1.00 (0.94, 1.06)**	27	0.81 (0.75, 0.87)	23	**0.95 (0.89, 1.02)**	0.02 (−0.06, 0.09)	0.610
PF 90°/s (Nm)	30	68.9 (63.7, 74.1)	29	70.4 (65.2, 76.6)	27	71.8 (66.4, 77.2)	23	77.0 (71.1, 82.9)	−3.7 (−11.6, 4.2)	0.358
PF 90°/s (Nm/kg)	30	0.56 (0.49, 0.62)	29	**0.78 (0.72, 0.85)**	27	0.62 (0.55, 0.69)	23	**0.82 (0.74, 0.89)**	0.03 (−0.11, 0.16)	0.702
PF 0° (Nm)	30	111.7 (102.1, 121.3)	29	106.8 (97.3, 116.4)	27	113.1 (103.1, 123.1)	23	120.2 (109.5, 130.8)	−12.0 (−23.4, −0.6)	0.039
PF 0° (Nm/kg)	30	0.91 (0.81, 1.01)	29	**1.19 (1.10, 1.30)**	27	0.91 (0.80, 1.01)	23	**1.27 (1.16, 1.38)**	−0.08 (−0.23, 0.07)	0.278
DF 90°/s (Nm)	26	8.2 (4.9, 11.5)	26	9.2 (5.9, 12.5)	23	6.0 (2.5, 9.5)	17	5.9 (4.7, 10.0)	1.2 (−5.2, 7.6)	0.715
DF 90°/s (Nm/kg)	26	0.07 (0.04, 0.10)	26	0.10 (0.07, 0.12)	23	0.05 (0.02, 0.09)	17	0.06 (0.02, 0.09)	0.03 (−0.03, 0.09)	0.340
DF 20° (Nm)	29	22.1 (19.3, 24.9)	28	18.3 (15.8, 21.5)	23	19.4 (16.3, 22.4)	23	17.6 (14.4, 20.8)	−2.0 (−5.8, 1.8)	0.300
DF 20° (Nm/kg)	29	0.18 (0.16, 0.21)	28	0.20 (0.17, 0.23)	23	0.16 (0.13, 0.18)	23	0.18 (0.15, 0.21)	−0.01 (−0.04, 0.03)	0.699
Physical function test										
3MWT (m/s)	30	1.09 (1.02, 1.16)	29	1.11 (1.04, 1.17)	28	1.04 (0.98, 1.11)	24	1.09 (1.02, 1.16)	−0.03 (−0.12, 0.06)	0.543
7.6MWT (m/s)	27	1.94 (1.84, 2.03)	29	**2.06 (1.97, 2.15)**	28	1.80 (1.71, 1.90)	24	**1.97 (1.87, 2.06)**	−0.04 (−0.15, 0.07)	0.451
2MWT (m/s)	30	1.57 (1.49, 1.65)	29	**1.73 (1.65, 1.81)**	28	1.52 (1.44, 1.60)	24	**1.67 (1.58, 1.76)**	0.01 (−0.06, 0.08)	0.786
STS5 (W)	30	508.5 (470.6, 546.3)	29	**428.3 (390.7, 466.0)**	28	491.1 (452.6, 529.7)	24	**443.7 (402.9, 484.6)**	−32.7 (−72.4, 6.9)	0.105
STS5 (W/kg)	30	4.09 (3.79, 4.39)	29	**4.70 (4.40, 5.00)**	28	3.90 (3.59, 4.20)	24	**4.61 (4.28, 4.94)**	−0.10 (−0.47, 0.27)	0.603
SCPT (W)	30	586.6 (554.4, 618.7)	28	**496.7 (464.0, 529.4)**	27	617.5 (584.1, 650.9)	22	**511.8 (474.4, 549.2)**	15.8 (−38.0, −69.6)	0.564
SCPT (W/kg)	30	4.77 (4.49, 5.05)	28	**5.53 (5.25, 5.81)**	27	4.91 (4.61, 5.20)	22	**5.35 (5.03, 5.67)**	0.31 (−0.10, 0.72)	0.141

*Note*: Table 2 presents the estimated means of participant characteristics at baseline and 12 months post treatment. Data are presented as means with 95% CI. The number of participants (*N*) at each time point is indicated in the table for both INT and CON. *p* values denote significant differences between the INT and CON, representing the interaction values from the mixed model. The interaction value indicates whether the development in the variable over time differs between the two groups, and bold text indicates within‐group differences from baseline to 12 months.

Abbreviations: 3MWT, 3‐min walk test; 7.6MWT, 7.6‐m walk test; 2MWT, 2‐min walk test; ALM, appendicular lean mass; CON, placebo group (*n* = 28); DF, dorsiflexion; F, female; HGS, handgrip strength; INT, zoledronic acid group (*n* = 31); KE, knee extension; KF, knee flexion; LBM, lean body mass; M, male; PF, plantar flexion; RYGB, Roux‐en‐Y gastric bypass; SCPT, stair climb power test; SE, shoulder elevation; SG, sleeve gastrectomy; STS5, 5‐repetition sit‐to‐stand test.

### 3.2 Muscle Strength

We did not observe any between‐group differences for isokinetic and isometric strength in the lower extremities (KE, KF, PF, and DF) in neither absolute or relative terms (Table 2), except for relative isometric KE, which favored INT (0.16 Nm/kg, 95% CI: 0.02–0.29, *p* = 0.021) and absolute isometric PF, which favored CON (−12.0 Nm/kg, 95% CI: −23.4 to −0.6, *p* = 0.039). For the upper extremities, a between‐group difference was observed in favor of the CON group for isometric shoulder strength (−16.8 Nm, 95% CI: −29.1 to −4.4, *p* = 0.008), while no difference was observed for handgrip strength.

### 3.3 Within‐Group Comparisons for Muscle Strength Outcomes

Within‐group comparisons showed that both groups declined in absolute knee extensor and flexor muscle strength across all contraction modalities (10.7%–15.4%, Figure 1 Panels B and C), while relative muscle strength improved across all contraction modalities (10.0%–22.0%, Figure 1, Panels B and C). For absolute PF muscle strength, both groups had preserved muscle strength across contraction modalities (Figure 1, Panel E), while relative muscle strength improved (31.0%–40.4%). For DF, muscle strength remained overall unchanged in both groups. For handgrip and shoulder strength, only INT experienced reductions of 4.6% and 15.6% (Figure 1, Panel H), respectively.

### 3.4 Physical Function

We did not observe any between‐group differences in habitual and maximal walking speed or endurance capacity (Table 2). Similarly, no between‐group differences were observed for lower limb muscle power in absolute or relative terms.

### 3.5 Within‐Group Comparisons for Physical Function Outcomes

Habitual walking speed was unchanged in both groups, while maximal walking speed increased by 6.3%–9.1% (Figure 1, Panel G). Furthermore, both groups improved their endurance capacity of ~10% (Figure 1, Panel G). Overall, for lower limb muscle power, both groups experienced a reduction in absolute power of 10%–17%, while relative power improved by 9%–16% (Figure 1, Panel D).

### 3.6 Physical Activity

At the 12‐month follow‐up, there were no between‐group differences for self‐reported total physical activity level (*p* = 0.243). However, for the subcategories, CON spent more time on moderate (*p* = 0.047) and high‐intensity (*p* = 0.048) activities compared with INT, while there was no difference in time spent walking (*p* = 0.377).

### 3.7 Between‐Surgery Type Comparison

For body composition, RYGB resulted in a greater fat mass reduction compared with SG (6.8 kg, 95% CI: 2.7–11.1, *p* < 0.001). There were no differences between RYGB and SG in reductions of ALM, LBM, or total body weight (Table S1 and Figure S1).

For all isokinetic muscle strength assessments, we observed between‐surgery differences, with SG showing greater declines in absolute muscle strength compared with RYGB (except for PF 90°/s), while RYGB had greater increases in relative muscle strength across all assessments compared with SG (Table S1 and Figure S1). For all isometric muscle strength assessments, there were no between‐surgery differences in absolute muscle strength, except for HGS, which favored RYGB (−2.4 kg, 95% CI: −4.4 to −0.3, *p* = 0.022). For relative muscle strength, we observed no between‐surgery differences for contraction modalities (Table S1 and Figure S1).

Regarding physical function, we did not observe a between‐surgery difference for any of the outcomes (Table S1 and Figure S1).

## 4 Discussion

In this randomized controlled trial, we investigated whether a single infusion of zoledronic acid 5 mg prior to bariatric surgery could be used as a prophylactic treatment to prevent the loss of muscle mass, muscle strength, and physical function following bariatric surgery. Our main finding was that zoledronic acid did not prevent the loss of LBM, muscle strength, or physical function compared with placebo. Therefore, we cannot confirm our hypothesis that a single infusion of zoledronic acid 5 mg would preserve LBM and muscle strength following bariatric surgery.

Zoledronic acid has been proposed to preserve muscle under conditions of muscle wasting [19, 20, 21, 22], although evidence remains inconsistent, with some studies failing to support this effect [24, 25, 26]. We did not observe differences between INT and CON for the majority of our outcomes (24 out of 27), indicating that preoperative administration of 5 mg zoledronic acid does not have protective effects against the loss of muscle mass and strength. Both groups experienced a similar loss of LBM and absolute muscle strength, along with similar improvements in relative muscle strength and physical function. These observations are in line with previous findings, showing similar declines in LBM and absolute strength, while relative muscle strength and physical function improve following surgery [2, 3, 4, 9, 10, 11].

As we did not perform a power analysis for the muscle and physical function outcomes prior to the trial, we cannot definitively rule out the possibility that the lack of between‐group differences was due to insufficient statistical power. However, we observed no between‐group differences in 24 out of 27 outcomes, with none approaching borderline significance (*p* values ranging from 0.126 to 0.900). Furthermore, in light of the potentially modest effects of bisphosphonates on muscle strength and physical function, we conducted two separate test–retest studies in a cohort meeting the criteria for bariatric surgery to determine the precision and the threshold that each outcome would need to surpass to be considered beyond the range of measurement error, defined by the standard error of measurement (SEM) [32, 34]. Overall, these studies demonstrated excellent reliability and low SEM across the applied assessments. In the present study, relative between‐group differences ranged from 0.3% to 6.2% (all nonsignificant), while SEM% thresholds ranged from 4.5% to 10.1%. None of the observed differences exceeded the threshold for any individual outcome, indicating that all effects fell within the range of measurement error. Therefore, our results do not indicate that a potential effect of zoledronic acid was masked by measurement error.

It may appear counterintuitive that zoledronic acid preserves bone density without also preserving lean mass. Bone and muscle are connected both mechanically and hormonally and often decline in parallel, especially with aging and catabolic conditions [38]. While increased muscle mass and strength can help maintain or increase bone mass through mechanical loading and myokine release, the reverse is less clear. Bone may influence muscle via osteokines and mineral release, but these signals are likely weaker and less direct than muscle's mechanical effect on bone [38]. Thus, treatment with antiresorptives like zoledronic acid may not preserve muscle mass, even when bone mass is maintained. For example, Haeri et al. and Blay et al. showed that zoledronic acid or risedronate increased bone mineral density in older women with and without osteoporosis but had no effect on age‐related decline in appendicular or total lean mass [27, 28]. Similarly, in our study, zoledronic acid prevented bone loss [18] but did not attenuate lean mass reduction. This suggests that targeting bone resorption alone is insufficient to preserve skeletal muscle in humans, despite promising preclinical findings.

If bisphosphonates have a muscle‐sparing effect, there are several reasons why we may not have observed it. Cytokines like TGF‐β and RANKL, which are embedded in the bone matrix, can promote muscle loss and impair muscle function when released during abnormal osteoclastic bone resorption [21, 29, 30]. Our previous findings showed that a single infusion of 5 mg zoledronic acid prevented bone loss in the spine and reduced bone loss in the hip but only partially suppressed bone resorption markers (85% vs. 166%) [18]. This suggests that the cytokine release was not fully prevented and may still have affected muscle tissue.

Bisphosphonates may also preserve muscle mass by downregulating atrophy pathways [20, 22]. Since zoledronic acid was administered an average of 26 ± 21 days preoperatively, it is unlikely that unbound bisphosphonate was available to act on muscle tissue postoperatively, as 60% is absorbed within 24 h and the rest is rapidly cleared from the bloodstream [39]. In animal studies, muscle preservation occurred with frequent bisphosphonate administration (two to three times per week) during muscle‐wasting conditions [20, 21, 29]. Translating these findings to humans is difficult, but one study showed that monthly risedronate for 6 months after bariatric surgery preserved ALM at 12 months, with a tendency for preserved midthigh CSA [23]. These findings should be interpreted with caution until larger trials from the same group may confirm risedronate's efficacy (NCT04922333) [40].

### 4.1 Nonuniform Loss of Muscle Strength Across Muscle Groups

We observed that muscle strength changes were not uniform across different muscle groups, suggesting that muscles respond differently to bariatric surgery. Thigh muscle strength (KE/KF) declined by 11%–18%, while calf muscles (DF/PF) showed no change. Shoulder elevation and handgrip strength declined modestly by ~9% (*p* = 0.017) and ~4% (*p* = 0.011) (data not shown). Variations in muscle fiber type composition may explain these differences, as Type II fibers (more abundant in thigh muscles) are more prone to atrophy after caloric restriction, malnutrition, and detraining [41, 42]. Additionally, it is possible that the thigh muscles are more affected by mechanical unloading and detraining as a result of the 25% weight reduction after bariatric surgery. This reduction in weight decreases the force required for activities like walking and stair climbing, leading to reduced stimulation of the thigh muscles, which may mimic a detraining condition. In contrast, the calf muscles may be less impacted by such unloading, as they are continuously active in balancing and stabilizing the body during movement and work closer to their maximal force capacity. Similarly, handgrip strength may be preserved as the hands are used frequently in daily activities, and minimal reductions in handgrip strength after bariatric surgery are not uncommon [43].

### 4.2 The Impact of the Type of Surgery on Muscle Strength and Body Composition

We observed that RYGB and SG had different effects on body composition and muscle function. While there was no difference in the reduction in body weight or ALM, RYGB led to a significantly greater decline in fat mass (6.8 kg corresponding to 42% greater reduction) compared with SG, aligning with a previous study [44]. RYGB showed less decline in isokinetic muscle strength (three out of four tests), while there were no differences in isometric muscle strength and muscle power. For relative muscle strength, RYGB showed greater improvements than SG in the isokinetic test, while there were no differences for isometric strength and power. The inconsistent results between isokinetic and isometric tests may be explained by the fact that these assessments measure different strength capabilities, with fast dynamic assessments being linked to rapid force generation due to the shorter time required to reach peak force (0.3–0.5 s vs. 3–5 s). Given that fat infiltration in skeletal muscle is closely linked to total fat mass [45], it is plausible that the greater fat mass reduction observed after RYGB (6.8 kg more than SG) also led to a greater reduction in fat infiltration. Reduced intramuscular fat has been associated with improved muscle strength and function [45], which could explain the greater improvements observed in dynamic strength following RYGB.

### 4.3 Strengths and Limitations

This study has several strengths. We employed a randomized controlled trial design, the gold standard for assessing intervention efficacy, ensuring that our findings are both reliable and unbiased. Additionally, we comprehensively assessed muscle and physical function across multiple domains using methods proven to be highly reliable in individuals who meet the criteria for bariatric surgery [32, 34].

There are several limitations to consider in this study. We acknowledge that the present results represent secondary analyses, and the study may therefore be underpowered to detect subtle differences between INT and CON. Further, our study design may make it difficult to determine whether bisphosphonates have muscle‐sparing effects. We administered zoledronic acid only once prior to surgery, which may not have been the optimal timing or provided in a sufficient dose to achieve a muscle‐sparing effect. Lower dosing and more frequent administration regimens, as used in other patient groups that are exposed to severe bone loss, could be an alternative treatment approach but require further investigation. Additionally, we did not measure key cytokines like TGF‐β and RANKL, which are hypothesized to contribute to muscle loss through bone resorption. Finally, the study population, consisting solely of individuals undergoing bariatric surgery, may limit the generalizability of our findings. It is uncertain whether bisphosphonates would be applicable to other populations experiencing muscle‐wasting conditions.

## 5 Conclusion

In conclusion, a single infusion of 5 mg zoledronic acid prior to bariatric surgery did not prevent the loss of muscle mass, muscle strength, or physical function compared with placebo.

## Author Contributions

Søren Gam, Stinus Gadegaard Hansen, Bibi Gram, Claus Bogh Juh, and Anne Pernille Hermann contributed substantially to the conception and design of the study. Søren Gam and Stinus Gadegaard Hansen were responsible for trial management. Søren Gam collected the majority of the data and performed the statistical analysis. Søren Gam wrote and edited the first draft of the manuscript under the supervision of Bibi Gram. All authors provided input to the manuscript. Prior to submission, all authors reviewed and approved the final version.

## Conflicts of Interest

Søren Gam reports receiving institutional grants for the present manuscript from Municipality Region of Southern Denmark, Aase og Ejner Danielsen fond, and Steno Diabetes Center Odense. He also reports support for attending meetings and travel from the University Hospital of Southern Denmark, Esbjerg Internationalization Fund for Travel Grants, William Demant Foundation, and PhD School at the University of Southern Denmark. Stinus Gadegaard Hansen reports receiving institutional grants for the present manuscript from A.P. Møller Fond, Karola Jørgensens Fond, and Municipality Region of Southern Denmark. Claus Bogh Juhl reports receiving honoraria for lectures and educational events from Novo Nordisk and serving on an advisory panel for the company. Additionally, he reports serving as the local principal investigator (PI) on studies sponsored by Novo Nordisk and Bayer. Anne Pernille Hermann reports receiving payment or honoraria for lectures, presentations, and participation in speaker bureaus from UCB and Amgen. She also reports support for attending meetings and travel, including conference fees and travel support from UCB. All other authors declare no conflicts of interest.

## Supporting information


**Figure S1:** The figure displays the within‐group relative changes in various body composition, muscle strength, and physical function metrics at 12 months post surgery. The light gray bars represent RYGB, while the dark gray bars represent SG. RYGB: Roux‐en‐Y gastric bypass; SG: sleeve gastrectomy; ALM: appendicular lean mass; LBM: lean body mass; HGS: handgrip strength; SE: shoulder elevation; KE: knee extension; KF: knee flexion; PF: plantar flexion; DF: dorsiflexion; STS5: 5‐repetition sit‐to‐stand test; SCPT: stair climb power test; 3MWT: 3‐min walk test; 7.6MWT: 7.6‐min walk test; 2MWT: 2‐min walk test. Statistical significance between baseline and 12 months within group is indicated by *p < 0.05, **p < 0.01, ***p < 0.001. Statistical interaction between RYGB and SG is indicated by ^#^ *p* < 0.05, ^##^ *p* < 0.01, ^###^ *p* < 0.001.

**Table S1:** Surgery effect estimates on body composition, muscle strength, and physical function. Table S1 presents the estimated means of participants' characteristics at baseline and 12 months post surgery. Data are presented as means with 95% confidence intervals (CI). The number of participants (*N*) at each time point is indicated in the table for both RYGB and SG. RYGB: Roux‐en‐Y gastric bypass; SG: sleeve gastrectomy; ALM: appendicular lean mass; LBM: lean body mass; HGS: handgrip strength; SE: shoulder elevation; KE: knee extension; KF: knee flexion; PF: plantar flexion; DF: dorsiflexion; STS5: 5‐repetition sit‐to‐stand test; SCPT: stair climb power test; 3MWT: 3‐min walk test; 7.6MWT: 7.6‐min walk test; 2MWT: 2‐min walk test. *p* values denote significant differences between RYGB and SG, representing the interaction values from the mixed model. The *P* value indicates whether the development in the variable over time differs between the surgery types. Bold text indicates within‐group differences from baseline to 12 months. * indicates between‐group differences at baseline.

## Data Availability

The data that support the findings of this study are available on request from the corresponding author. The data are not publicly available due to privacy or ethical restrictions.
