# Supplementary figures and images for: Effect of Zoledronic Acid on Skeletal Muscle After Bariatric Surgery: A Secondary Analysis From a Randomized Controlled Trial
Source: Obesity (Silver Spring). 2025 Nov 2;34(1):76–87. doi: 10.1002/oby.70062 (PMC12724060; doi:10.1002/oby.70062)

1A

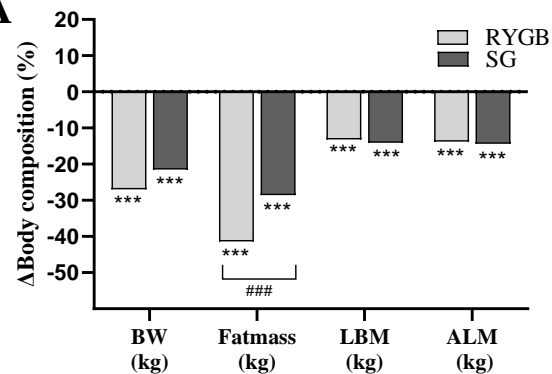

1B

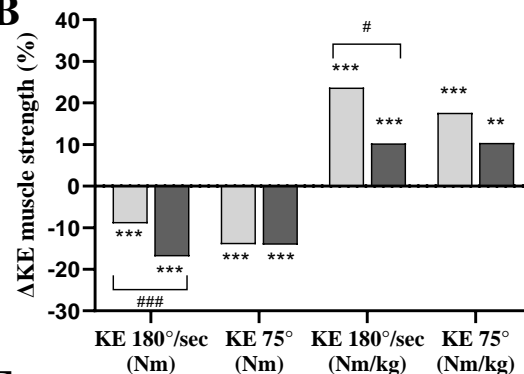

1C

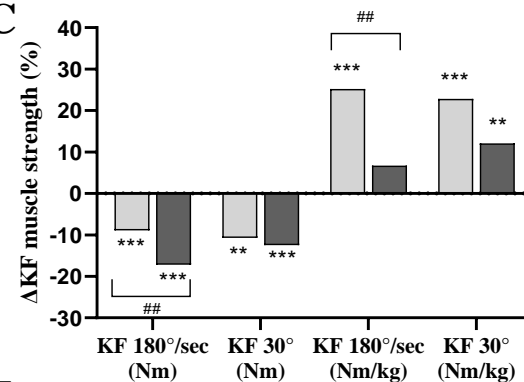

1D

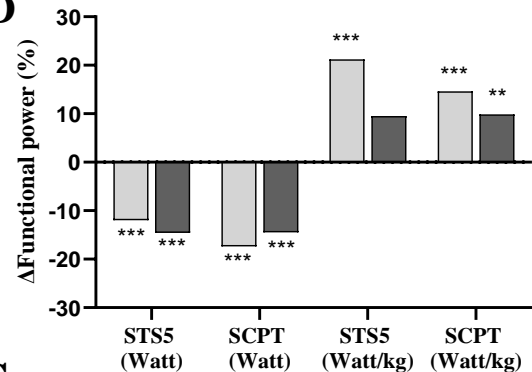

1E

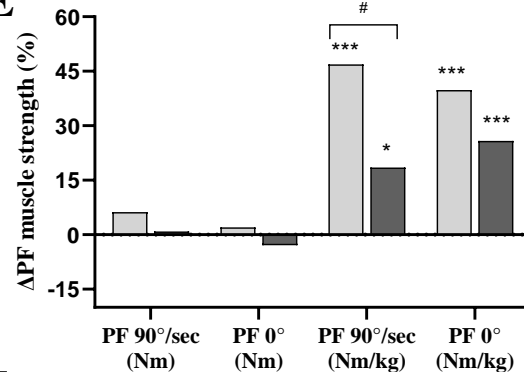

1F

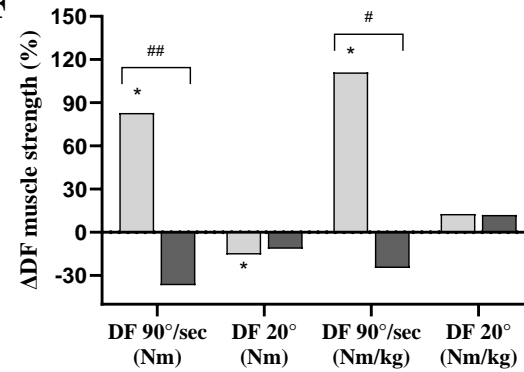

1G

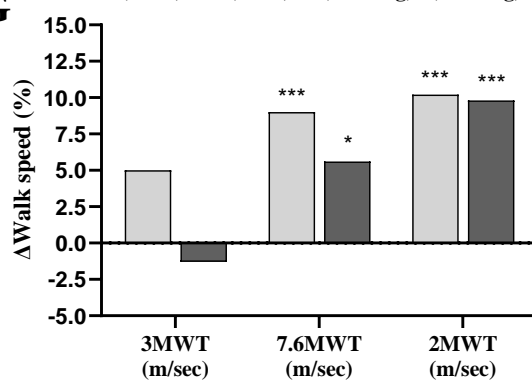

1H

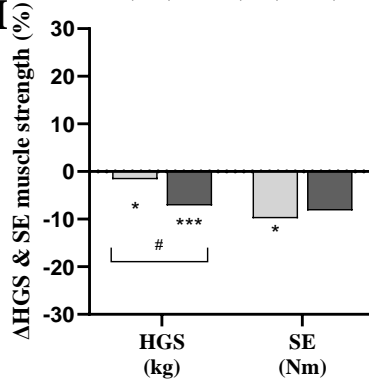

Supplement: Supplementary file 1 — Figure S1: The figure displays the within‐group relative changes in various body composition, muscle strength, and physical function metrics at 12 months post surgery. The light gray bars represent RYGB, while the dark gray bars represent SG. RYGB: Roux‐en‐Y gastric bypass; SG: sleeve gastrectomy; ALM: appendicular lean mass; LBM: lean body mass; HGS: handgrip strength; SE: shoulder elevation; KE: knee extension; KF: knee flexion; PF: plantar flexion; DF: dorsiflexion; STS5: 5‐repetition sit‐to‐stand test; SCPT: stair climb power test; 3MWT: 3‐min walk test; 7.6MWT: 7.6‐min walk test; 2MWT: 2‐min walk test. Statistical significance between baseline and 12 months within group is indicated by *p < 0.05, **p < 0.01, ***p < 0.001. Statistical interaction between RYGB and SG is indicated by # p < 0.05, ## p < 0.01, ### p < 0.001. [file OBY-34-76-s002.pdf]
