# Supplementary material for: Effect of Zoledronic Acid on Skeletal Muscle After Bariatric Surgery: A Secondary Analysis From a Randomized Controlled Trial
Source: Obesity (Silver Spring). 2025 Nov 2;34(1):76–87. doi: 10.1002/oby.70062 (PMC12724060; doi:10.1002/oby.70062)
Supplement: Supplementary file 2 — Table S1: Surgery effect estimates on body composition, muscle strength, and physical function. Table S1 presents the estimated means of participants' characteristics at baseline and 12 months post surgery. Data are presented as means with 95% confidence intervals (CI). The number of participants (N) at each time point is indicated in the table for both RYGB and SG. RYGB: Roux‐en‐Y gastric bypass; SG: sleeve gastrectomy; ALM: appendicular lean mass; LBM: lean body mass; HGS: handgrip strength; SE: shoulder elevation; KE: knee extension; KF: knee flexion; PF: plantar flexion; DF: dorsiflexion; STS5: 5‐repetition sit‐to‐stand test; SCPT: stair climb power test; 3MWT: 3‐min walk test; 7.6MWT: 7.6‐min walk test; 2MWT: 2‐min walk test. p values denote significant differences between RYGB and SG, representing the interaction values from the mixed model. The P value indicates whether the development in the variable over time differs between the surgery types. Bold text indicates within‐group differences from baseline to 12 months. * indicates between‐group differences at baseline. [file OBY-34-76-s001.docx]

| **Table S1:** Surgery effect estimates on body composition, muscle strength, and physical function | | | | | | | | | | | | |
| --- | --- | --- | --- | --- | --- | --- | --- | --- | --- | --- | --- | --- |
|  |  | **SG (N = 19, F/M = 15/4 )** | | |  |  | **RYGB (N =40, F/M = 27/13)** | | |  | **Between surgery differences** | |
|  |  | **Baseline** |  | **12 Month** |  |  |  |  |  |  | **SG-RYGB** | |
| **Body composition** | N | Mean (CI) | N | Mean (CI) |  | N | Mean (CI) | N | Mean (CI) |  | Mean (CI) | P |
| Weight (kg) | 19 | 129.4 (122.3, 136.6) | 18 | **101.6 (94.5, 108.8)** |  | 40 | 120.7 (115.7, 125.6) | 35 | **88.1 (83.1, 93.2)** |  | 4.7 (-0.7, 10.2) | 0.090 |
| BMI (kg/m^2^) | 19 | 43.1 (40.8, 45.4) | 18 | **34.0 (31.6, 36.3)** |  | 40 | 41.9 (40.3, 43.5) | 35 | **30.6 (28.9, 32.2)** |  | 2.2 (0.4, 4.1) | 0.019 |
| Fat mass (kg) | 19 | 56.3 (51.6, 60.9) | 18 | **40.2 (35.5, 44.9)** |  | 40 | 55.3 (52.1, 58.5) | 35 | **32.4 (29.1, 35.7)** |  | 6.8 (2.7, 11.1) | 0.001 |
| Fat percentage (%) | 19 | 43.7 (41.7, 45.7) | 18 | **38.6 (36.6, 40.7)** |  | 40 | 45.6 (44.2, 47.0) | 35 | **35.7 (34.2, 37.1)** |  | 4.8 (2.3, 7.4) | 0.001 |
| LBM (kg)* | 19 | 68.7 (66.0, 71.5) | 18 | **59.0 (56.3, 61.8)** |  | 40 | 62.0 (60.1, 63.9) | 35 | **53.8 (51.9, 55.7)** |  | -1,9 (-4.0, 0.2) | 0.127 |
| ALM (kg)* | 19 | 31.4 (30.0, 32.9) | 18 | **26.9 (25.4, 28.4)** |  | 40 | 28.3 (27.3, 29.3) | 35 | **24.4 (23.4, 25.5)** |  | -0.6 (-1.7, 0.5) | 0.266 |
| **Muscle strength** |  |  |  |  |  |  |  |  |  |  |  |  |
| HGS (kg) | 19 | 42.0 (39.3, 44.7) | 18 | **39.0 (36.3, 41.7)** |  | 39 | 36.9 (35.0, 38.77) | 35 | 36.3 (34.3, 38.2) |  | -2.4 (-4.4, -0.3) | 0.022 |
| SE (Nm)* | 19 | 132.3 (119.9, 144.7) | 16 | 121.5 (108.5, 134.6) |  | 39 | **100.8 (92.2, 109.4)** | **35** | **90.9 (82.0, 99.9)** |  | -1.0 (-15.1, 13.1) | 0.894 |
| KE 180°/s (Nm)* | 19 | 152.8 (141.1, 164.4) | 18 | **126.9 (115.2, 138.6)** |  | 38 | 120.7 (112.5, 128.9) | 34 | **109.8 (101.4, 118.4)** |  | -14.9 (-25.0, -4.9) | 0.004 |
| KE 180°/s (Nm/kg)* | 19 | 1.14 (1.06, 1.22) | 18 | **1.25 (1.17, 1.33)** |  | 38 | 1.00 (0.95, 1.06) | 34 | **1.24 (1.18, 1.30)** |  | -0.12 (-0.22, -0.03) | 0.013 |
| KE 75° (Nm)* | 19 | 261.4 (245.1, 277.8) | 18 | **224.5 (208.0, 241.0)** |  | 38 | 220.6 (209.0, 232.1) | 34 | **187.1 (170.0, 204.2)** |  | -6.0 (-21.1, 9.1) | 0.435 |
| KE 75° (Nm/kg) | 19 | 2.01 (1.86, 2.17) | 18 | **2.22 (2.06, 2.38)** |  | 38 | 1.81 (1.70, 1.92) | 34 | **2.13 (2.02, 2.24)** |  | -0.11 (-0.25, 0.04) | 0.138 |
| KF 180°/s (Nm)* | 19 | 77.9 (72.3, 83.6) | 18 | **64.5 (58.8, 70.2)** |  | 38 | 61.2 (57.2, 65.2) | 34 | **55.8 (51.7, 59.9)** |  | -8.0 (-12.9, -3.0) | 0.002 |
| KF 180°/s (Nm/kg)* | 19 | 0.60 (0.55, 0.64) | 18 | 0,64 (0.59, 0.68) |  | 38 | 0.50 (0.47, 0.53) | 34 | **0.63 (0.60, 0.66)** |  | -0.09 (-0.14, -0.04) | 0.001 |
| KF 30° (Nm)* | 19 | 122.5 (112.2, 132.8) | 18 | **104.0 (93.6, 114.5)** |  | 38 | 94.4 (88.3, 100.5) | 34 | **84.3 (78.0, 90.6)** |  | -4.8 (-13.8, 4.2) | 0.294 |
| KF 30° (Nm/kg)* | 19 | 0.92 (0.85, 0.99) | 18 | **1.03 (0.96, 1.11)** |  | 38 | 0.77 (0.72, 0.82) | 34 | **0.95 (0.90, 1.00)** |  | -0.06 (-0.14, 0.01) | 0.096 |
| PF 90°/s (Nm) | 19 | 77.5 (71.1, 83.9) | 18 | 78.1 (71.6, 84.5) |  | 38 | 66.7 (62.2, 71.2) | 34 | 70.8 (66.1, 75.6) |  | -6.3 (-15.9, 3.2) | 0.407 |
| PF 90°/s (Nm/kg)* | 19 | 0.66 (0.58, 0.74) | 18 | **0.78 (0.70, 0.87)** |  | 38 | 0.55 (0.49, 0.61) | 34 | **0.81 (0.75, 0.87)** |  | -0.14 (-0.27, -0.00) | 0.049 |
| PF 0° (Nm) | 19 | 122.2 (110.5, 134.0) | 18 | 118.7 (106.7, 130.6) |  | 38 | 107.5 (99.1, 115.8) | 34 | 109.6 (101.0, 118.2) |  | -5.7 (-18.1, 6.6) | 0.363 |
| PF 0° (Nm/kg) | 19 | 0.94 (0.82, 1.06) | 18 | 1.19 (1.06, 1.31) |  | 38 | 0.89 (0.81, 0.98) | 34 | **1.25 (1.16, 1.34)** |  | -0.11 (-0.26, 0.04) | 0.149 |
| DF 90°/s (Nm)* | 18 | 11.0 (7.2, 14.9) | 18 | 7,0 (3.2, 10.8) |  | 31 | 4.8 (1.8, 7.7) | 26 | **8.7 (5.5, 11.9)** |  | -8.0 (-14.1, -1.9) | 0.010 |
| DF 90°/s (Nm/kg)* | 18 | 0.09 (0.06, 0.13) | 18 | 0.07 (0.03, 0.10) |  | 31 | 0.04 (0.01, 0.07) | 26 | **0.09 (0.06, 0.11)** |  | -0.07 (-0.12, -0.01) | 0.016 |
| DF 20° (Nm)* | 18 | 25.6 (22.2, 29.1) | 17 | 22.7 (19.2, 26.2) |  | 34 | 18.4 (15.9, 20.9) | 34 | **15.6 (13.0, 18.1)** |  | -0.1 (-4.1, 3.9) | 0.973 |
| DF 20° (Nm/kg)* | 18 | 0.20 (0.17, 0.23) | 17 | 0.22 (0.19, 0.26) |  | 34 | 0.15 (0.13, 0.18) | 34 | 0.17 (0.15, 0.20) |  | 0.00 (-0.03, 0.04) | 0.805 |
| **Physical function test** |  |  |  |  |  |  |  |  |  |  |  |  |
| 3MWT (m/sec)* | 19 | 1.2 (1.1, 1.3) | 18 | 1.2 (1.1, 1.2) |  | 39 | 1.0 (1.0, 1.1) | 35 | 1.1 (1.0, 1.1) |  | -0.1 (-0.2, 0.0) | 0.170 |
| 7.6MWT (m/sec)* | 18 | 2.0 (1.9, 2.2) | 18 | 2.1 (2.0, 2.2) |  | 37 | 1.8 (1.7, 1.9) | 35 | **2.0 (1.9, 2.0)** |  | 0.0 (-0.2, 0.1) | 0.419 |
| 2MWT (m/sec) | 19 | 1.6 (1.52, 1.71) | 18 | **1.8 (1.68, 1.87)** |  | 39 | 1.5 (1.46, 1.58) | 35 | **1.7 (1.6, 1.7)** |  | -0.0 (-0.1, 0.1) | 0.921 |
| STS5 (watt)* | 19 | 592.9 (547.1, 638.8) | 18 | **506.3 (460.0, 552.5)** |  | 39 | 453.9 (422.0, 485.8) | 35 | **399.5 (366.6, 432.4)** |  | -32.2 (-74.0, -9.6) | 0.131 |
| STS5 (watt/kg)* | 19 | 4.6 (4.20, 4.93) | 18 | 5.0 (4.63, 5.37) |  | 39 | 3.7 (3.45, 3.96) | 35 | **4.5 (4.2, 4.8)** |  | -0.4 (-0.7, -0.0) | 0.067 |
| SCPT (watt)* | 19 | 666.0 (626.4, 705.5) | 18 | **569.5 (529.2, 609.9)** |  | 38 | 567.4 (539.5, 595.3) | 32 | **468.5 (438.4, 498.7)** |  | 2.4 (-53.8, 58.6) | 0.933 |
| SCPT (watt/kg) | 19 | 5.1 (4.8, 5.5) | 18 | 5.7 (5.3, 6.0) |  | 38 | 4.7 (4.4, 4.9) | 32 | **5.4 (5.1, 5.6)** |  | -0.2 (-0.6, 0.9) | 0.440 |
|  | | | | | | | | | | | | |

**Table S1 Caption
Table S1** presents the estimated means of participants' characteristics at baseline and 12 months post-surgery. Data are presented as means with 95% confidence intervals (CI). The number of participants (N) at each time point is indicated in the table for both RYGB and SG. Abbreviations: RYGB: Roux-en-Y gastric bypass; SG: Sleeve gastrectomy; BMI: body mass index; ALM: appendicular lean mass; LBM: lean body mass; HGS: hand grip strength; SE: shoulder elevation; KE: knee extension; KF: knee flexion; PF: plantar flexion; DF: dorsiflexion; STS5: 5 times sit-to-stand test; SCPT: stair climb power test; 3MWT: 3-minute walk test; 7.6MWT: 7.6-meter walk test; 2MWT: 2-minute walk test. P values denote significant differences between RYGB and SG, representing the interaction values from the mixed model. The P value indicates whether the development in the variable over time differs between the surgery types. Bold text indicates within-group differences from baseline to 12 months. * indicates between-group differences at baseline.
